# Neonatal Encephalopathy Is Associated With Altered IL-8 and GM-CSF Which Correlates With Outcomes

**DOI:** 10.3389/fped.2020.556216

**Published:** 2021-02-08

**Authors:** Deirdre U. Sweetman, Tammy Strickland, Ashanty M. Melo, Lynne A. Kelly, Chike Onwuneme, William R. Watson, John F. A. Murphy, Marie Slevin, Veronica Donoghue, Amanda O'Neill, Eleanor J. Molloy

**Affiliations:** ^1^Neonatology, National Maternity Hospital, Dublin, Ireland; ^2^National Children's Research Centre, Dublin, Ireland; ^3^Paediatrics, Royal College of Surgeons in Ireland, Dublin, Ireland; ^4^Discipline of Paediatrics, Trinity College Dublin, Dublin, Ireland; ^5^Trinity Translational Medicine Institute, St James Hospital, Dublin, Ireland; ^6^UCD School of Medicine and Medical Sciences, University College Dublin, Dublin, Ireland; ^7^Radiology Department, Children's University Hospital, Dublin, Ireland; ^8^Childrens University Hospital (CHI) at Tallght, Tallaght University Hospital, Dublin, Ireland; ^9^Paediatrics, Coombe Women's and Infant's University Hospital, Dublin, Ireland; ^10^Neonatology, Children's Health Ireland at Crumlin, Dublin, Ireland

**Keywords:** outcomes, cytokines, therapeutic hypothermia, neonatal brain injury, perinatal asphyxia, hypoxic-ischemic encephalopathy

## Abstract

**Aim:** To investigate the relationship between cytokines associated with innate immune cell activation and brain injury and outcome in infants with NE compared to neonatal controls.

**Methods:** Serum and CSF biomarkers associated with activated neutrophils and monocytes [Interleukin-8 (IL-8) and Granulocyte-Macrophage-Colony-Stimulating-Factor (GM-CSF)] were serially measured using duplex immunoassays on days 1, 3 and 7 in term newborns with NE and controls. Results were compared to grade of encephalopathy, seizures, MRI brain imaging, mortality and Bayley Score of Infant and Toddler Development (Bayley-III) at 2 years of age.

**Results:** Ninety-four infants had serum samples collected with 34 CSF samples. NE Grade II/III was significantly associated with elevated on day 2 serum IL-8. Mortality was best predicted by elevated day 1 IL-8. GM-CSF was initially elevated on day 1 and abnormal MRI imaging was associated with decreased day 2 GM-CSF. Elevated GM-CSF at day of life 6–7 correlated negatively with composite cognitive, language and motor Bayley-III scores at 2 years.

**Conclusion:** Moderate or severe NE and mortality was associated with elevated IL-8. Day 2 GM-CSF could predict abnormal MRI results in NE and Bayley-III. Therefore, these cytokines are altered in NE and may predict early outcomes and further implicate inflammatory processes in NE.

## Introduction

Neonatal encephalopathy (NE) can result in long-term neurodevelopmental impairment in term infants. Therapeutic hypothermia (TH) is the only standard treatment but morbidity remains elevated at 50% (1–3). Systemic inflammation and a dysregulated immune response are features of NE (4, 5). Elevated leukocytes are associated with adverse neurodevelopmental outcome in infants with NE (6). We have previously reported the alteration of vascular endothelial growth factor (VEGF) and erythropoietin (Epo), two cytokines associated with hypoxia in NE infants, and the use of these cytokines as markers of severity of hypoxia-ischemia and brain injury (7).

Infants with NE also present systemic inflammation, therefore we were interested in cytokines associated with innate immune cell activation such as Interleukin-8 (IL-8) and granulocyte-macrophage colony-stimulating factor (GM-CSF). IL-8 and GM-CSF stimulate neutrophils and monocytes in both adults and neonates (8). IL-8 is a chemotactic cytokine that mainly facilitates neutrophil recruitment and activation during immunological responses at the site of inflammation (9). IL-8 concentrations in the serum of neonates with perinatal asphyxia are significantly higher than levels in control newborns on day 1 of life and has been suggested as a suitable candidate biomarker in NE (10–12).

GM-CSF is a member of the βc family of glycoprotein cytokines that has potent effects in stimulating the proliferation, maturation and function of haematopoietic cells as well as regulating multiple biological processes such as native and adaptive immunity, inflammation, normal and autoimmunity but is also thought to exert biological effects on non-haematopoietic cells (13). Imbalance of GM-CSF may induce chronic inflammation and brain inflammation (14, 15). Spath et al. described that the excess production of GM-CSF induces reactive oxygen species (ROS) by brain-infiltrating phagocytes leading to spontaneous brain inflammation and neurological disease (14).

We hypothesized that infants with NE may have altered serum and cerebrospinal fluid (CSF) cytokines associated with leukocyte activation (GM-CSF and IL-8) and may reflect severity of brain injury in NE. The objective of this study was to investigate the relationaship between serum biomarkers and outcome suh as severity of encephalopathy, seizures, MRI brain, mortality and neurodevelopment in infants with NE.

## Materials and Methods

### Ethical Approval

Ethical committee approval was received from the National Maternity Hospital, Dublin, a tertiary referral, University-affiliated Maternity hospital in 2011. In all cases written informed consent was taken from parents of infants enrolled in the study.

### Patient Groups

We prospectively recruited infants with NE and neonatal controls as previously described (7, 16). The following study groups were enrolled: **Neonatal Controls:** Serum samples from term healthy control infants following normal delivery with normal Apgar scores, normal neurological examination and postnatal course; **Neonatal Encephalopathy:** Infants were divided into subgroups according to the grade of clinical encephalopathy according to the classification of Sarnat and Sarnat as follows: (a) **NE 0/I**: infants who required resuscitation following delivery with no neurological signs or mild encephalopathy; (b) **NE II/III**: moderate/severe encephalopathy (17). Infants with congenital abnormalities or evidence of maternal substance abuse were excluded.

All infants had serial cranial ultrasounds performed within the first 24 h of life and those with NE had an MRI brain within the first 10 days of life. MRIs were scored and reported independently by a pediatric radiologist as either “normal” or “abnormal” and classified with the Barkovich score (18). Seizures were diagnosed clinically based on recognition by nursing/medical staff of abnormal paroxysmal, repetitive and stereotypical events. Infants with NE also had continuous video EEG monitoring or aEEG monitoring over the first 3 days of life and were graded in a blinded fashion as having a “normal” or “abnormal” EEG by the electrophysiologist (GB) (19).

NE infants were followed up at 18–24 months of age and had a Bayleys Score of Infant Development III performed by a developmental psychologist (MS). Composite cognitive, language and motor developmental scores were grouped as normal (>90 in each category) or at risk or abnormal (<90 in each category).

### Magnetic Resonance Imaging

All infants with NE were examined on a 1.5T scanner and all were examined before day 10 of life. The sequences used were: Diffusion with ADC Map, T1 in axial and sagittal planes, T2 in axial plane, gradient echo and spectroscopy.

### Serum and CSF Sampling

Serum samples were collected from infants at risk of NE at 1, 2, 3, and 7 days of life. At each time point 1.2 mL of serum were collected either from a peripheral venous or arterial catheter sample and were centrifugated (3,000 rpm at 4°C × 5 min) and the supernatant was stored at −80°C until batch cytokine analysis was carried out. CSF samples were similarly centrifugated and the supernatant stored at −80°C. Frozen serum and CSF samples were thawed at room temperature, then kept on ice until ready to assay (7).

### Cytokine Analysis

Interleukin-8 and GM-CSF were determined by immunoassay using commercial kits (Human Ultra-Sensitive IL-8 and Human Ultra-Sensitive GM-CSF®, Meso Scale Diagnostic, MA, USA). All blockers and wash buffers were prepared and validated according to manufacturer's guidelines. The assays employed a sandwich immunoassay format where capture antibodies were coated in a patterned array on the bottom of the wells of a singleplex plate. The samples were analyzed on these pre-coated plates according to the manufacturer's instructions using a SECTOR® Imager where a voltage applied to the plate electrodes causes the captured labels to emit light and the intensity of emitted light provides a quantitative measure of analytes in the sample. Reproducibility was good with calculated concentration %CV <5% for standards in the quantitative range of the assays (7).

### Statistical Analysis

Statistical analysis was carried out using the PASW statistical package version 18 (www.ibm.com/SPSS_Statistics). Significance was assumed for values of *p* < 0.05. We divided the timing of biomarker sampling into 5 blocks; day 1 (D1), day 2 (D2), day 3 (D3), days 4 and 5 (D4–5) and days 6, 7, and 8 (D6–8). The majority of the serum samples were taken over days 1–3 of life. A score of 3 or 4 for the combined Basal Ganglia/Watershed Barkovich score was coded as severe brain injury. Histogram analysis of the serum and CSF biomarker data revealed a non-normal distribution. Therefore medians (IQRs) were employed to describe the data and non-parametric tests, the Mann-Whitney *U* and Kruskal-Wallis test were used for comparative analysis with outcomes of TH, seizure occurence, grade of NE, MRI brain result, mortality. Bayley-III outcomes at 18–24 months of age were correlated with serum and CSF biomarker concentrations in the first week of life via Spearman correlation. Receiver Operating Characteristic (ROC) curves and cut-off values were calculated for all outcomes.

## Results

### Clinical Outcomes

The study enrolled a total of 94 term neonates including controls (*n* = 12) and infants exposed to perinatal asphxia with NE 0-III (*n* = 82). There were serum samples from 94 neonates (*n* = 247 total serum samples) and 34 neonates with NE had CSF samples collected during their NICU admission. The neonatal controls had a mean gestational age of 39.4 ± 1.2 weeks and birth weight of 3.5 ± 0.4 kg with 6 males. All were born by spontaneous vaginal delivery with Apgar scores at both 1 and 5 min of 9 ± 1 and had normal neurological examinations. There were no significant differences between controls and NE cases with regard to gestational age, birth weight, gender or outborn status. Infants with NE were significantly more likely to be delivered by lower segment cesarean section or instrumental delivery and had significantly lower Apgar scores at 1 and 5 min compared to controls.

The decision to perform a lumbar puncture was made by the treating neonatologist based on a clinical suspicion of sepsis/meningitis. Thirty-nine infants required TH in accordance with the TOBY (Total Body Hypothermia for Neonatal Encephalopathy) criteria (20) and were treated for 72 h duration and 4 infants died. The grades of encephalopathy according to Sarnat and Sarnat (17) were as follows: Infants exposed to perinatal asphyxia but with no neurological signs (denoted as grade 0, *n* = 6); Mild NE (grade I, *n* = 23); Moderate NE (grade II, *n* = 42); Severe NE (grade III, *n* = 11). The NE group II/III were significantly more likely to be outborn, have lower 1, 5, and 10 min Apgar scores, have clinical seizures, undergo TH, have abnormal MRI brain imaging and had significantly lower admission pH, admission bicarbonate and larger base excess values compared to NE group 0/I. However, there were no significant differences between the NE groups 0/I and II/III with regard to gestational age, birth weight, gender, mode of delivery, multiplicity, mortality, cord pH, cord base excess and cord lactate values, as previously described (7).

### Serum Biomarker Data

GM-CSF and IL-8 concentration were measured on serum samples from NE infants and controls. GM-CSF levels were not significantly different in NE patients vs. controls during first week of life. On the contrary, IL-8 was significantly higher on day 1 (*p* = 0.0017), 2 (*p* = 0.0048), and 3 (*p* = 0041) in NE patients compared to controls. IL-8 concentration in NE infants serum was significantly lower on day 7 compared to day 1 (*p* = 0.0005), day 2 (*p* = 0.0012), and day 3 (*p* = 0.0017) of life (Table 1).

**Table 1:** GM-CSF and IL-8 concentrations in controls and NE infants over the first week of life.

**Cytokine**	**Control**	**NE D1**	**NE D2**	**NE D3**	**NE D6–8**
GM-CSF (pg/mL)	0.56 (0.08)	0.59 (0.05)	0.62 (0.06)	1.06 (0.17)	0.65 (0.09)
IL8 (pg/mL)	35.86 (7.0)	133.81** (23.3)	137.53** (29.2)	149.07** (30.2)	49.86** (10.1)

Serum concentrations of GM-CSF and IL-8 in NE patients (n = 82) expressed as pg/mL (day 1–7) vs. controls (n = 12) were measured by duplex cytokine analysis in the first week of life. (**p < 0.01 using the Mann–Whitney test).

Infants who underwent TH had significantly elevated IL-8 levels on day 3 (*p* = 0.008) (Table 2a). GM-CSF concentration was not altered. Grade II/III NE was significantly associated with elevated serum IL-8 levels on days 2 and 6–8 (*p* = 0.02, cut-off level 34.23 pg/mL) (Table 2a). Seizures were not significantly associated with any of the serum or CSF biomarkers investigated, whereas mortality was associated with high concentration of GM-CSF (*p* = 0.035) and IL-8 (*p* = 0.01) on day 1 (Table 2b).

**Table 2:** Associations between serum biomarker values of cases infants and outcome measures of (a) TH and NE grade and (b) MRI brain and mortality.

**Biomarker**	**Day of life**	**TH**			**NE Grade**		
		**Yes**	**No**	***p***	**0/I**	**II/III**	***p***
**(a)**							
IL-8	**2**	65.7 (47.3–142.2)	37.7 (21.8–267.5)	0.12	33.5 (21.8–59.6)	75.3 (44.4–232.6)	0.02
	**3**	68.4 (46.5–153.2)	32.5 (17.7–85.9)	0.008	32.5 (18.3–59.1)	66 (36.7–145)	0.07
	**6–8**	30.5 (21.8–45.9)	44.9 (28.3–124.4)	0.26	9.1^x^	35.7 (25.2–49.3)	0.01
**(b)**								
**Biomarker**	**Day of life**	**MRI**			**Survival**		
		**Abnormal**	**Normal**	***p***	**No**	**Yes**	***p***
GM-CSF	**1**	0.48 (0.29–0.67)	0.36 (0.26–0.73)	0.63	1.21 (0.54–1.56)	0.41 (0.29–0.74)	0.035
	**2**	0.39 (0.2–0.63)	0.7 (0.41–1.19)	0.001	0.31 (0.19–0.48)	0.54 (0.35–0.81)	0.13
	**3**	0.56 (0.36–0.97)	1.21 (0.64–1.47)	0.01	0.54^x^	0.69 (0.45–1.23)	0.33
IL−8	**1**	74.8 (27–444.5)	86 (40.6–154.2)	0.91	1834.2 (283.2–4932.3)	72.3 (35.6–181.7)	0.01
	**3**	81.6 (23.7–253.3)	56.1 (38.9–94.6)	0.69	300^x^	55.6 (32.7–112.8)	0.05

*Medians and IQRs of (a) TH vs. no TH and grade of NE and (b) normal vs. abnormal MRI and survival in NE are listed in each column, followed by a p-value for each comparison; Days without significant results excluded; x = no interquartile range available due to small sample numbers. All analysis used the Mann-Whitney U-test for comparisons of non-parametric data*.

### Magnetic Resonance Imaging Findings

MRI brain scans were performed on 66 infants (patients with normal MRI *n* = 35). Barkovich scores were available for the MRI brain scans of 64 infants with NE with the following results: Basal Ganglia (BG) score: 1 (*n* = 3);2 (*n* = 2); 3 (*n* = 2); 4 (*n* = 6). Watershed (W) score=1 (*n* = 7);2 (*n* = 3); 4 (*n* = 3); 5 (*n* = 3). Combined Basal Ganglia/Watershed (BG/W) score=1 (*n* = 5); 2 (*n* = 10); 3 (*n* = 6); 4 (*n* = 1). There were significantly higher levels of serum GM-CSF on day 3 in infants with BG/W scores of 3/4 compared to those who scored 1/2 [1 (0.5–2.8) vs. 0.4 (0.2–0.7) pg/mL; *p* = 0.03]. GM-CSF levels increased over time in neonates with normal MRI brain. Abnormal MRI brain imaging was significantly associated with decreased GM-CSF levels on day 2 (*p* = 0.001) and day 3 (*p* = 0.01) compared to normal MRI brain image, whereas IL-8 concentration was not altered (Table 2b).

### Neurodevelopmantal Followup (Bayley-III)

Neurodevelopmental assessments at 18–24 months of age were performed on 43 infants of the 53 infants with NE II/III originally recruited. Composite cognitive, language and motor Bayley-III scores were calculated and divided into normal (Bayley III>90) and abnormal (Bayley III <90) categories. 21, 30, and 12% demonstrated cognitive, language and motor delay, respectively. The diagnoses were as follows: 7 (16%) were diagnosed with a disability, including cerebral palsy (CP. *n* = 1) and autism (*n* = 2). Bayley scores <90 in either cognition, motor, or language were detected in 17 (40%): 14 (32%) language, 7 (16%) cognitive, and 6 (14%) motor domain. Infants with disability had more abnormalities on discharge examination and brain MRI, with longer hospital stay (*p* < 0.001).

**Figure 1:**
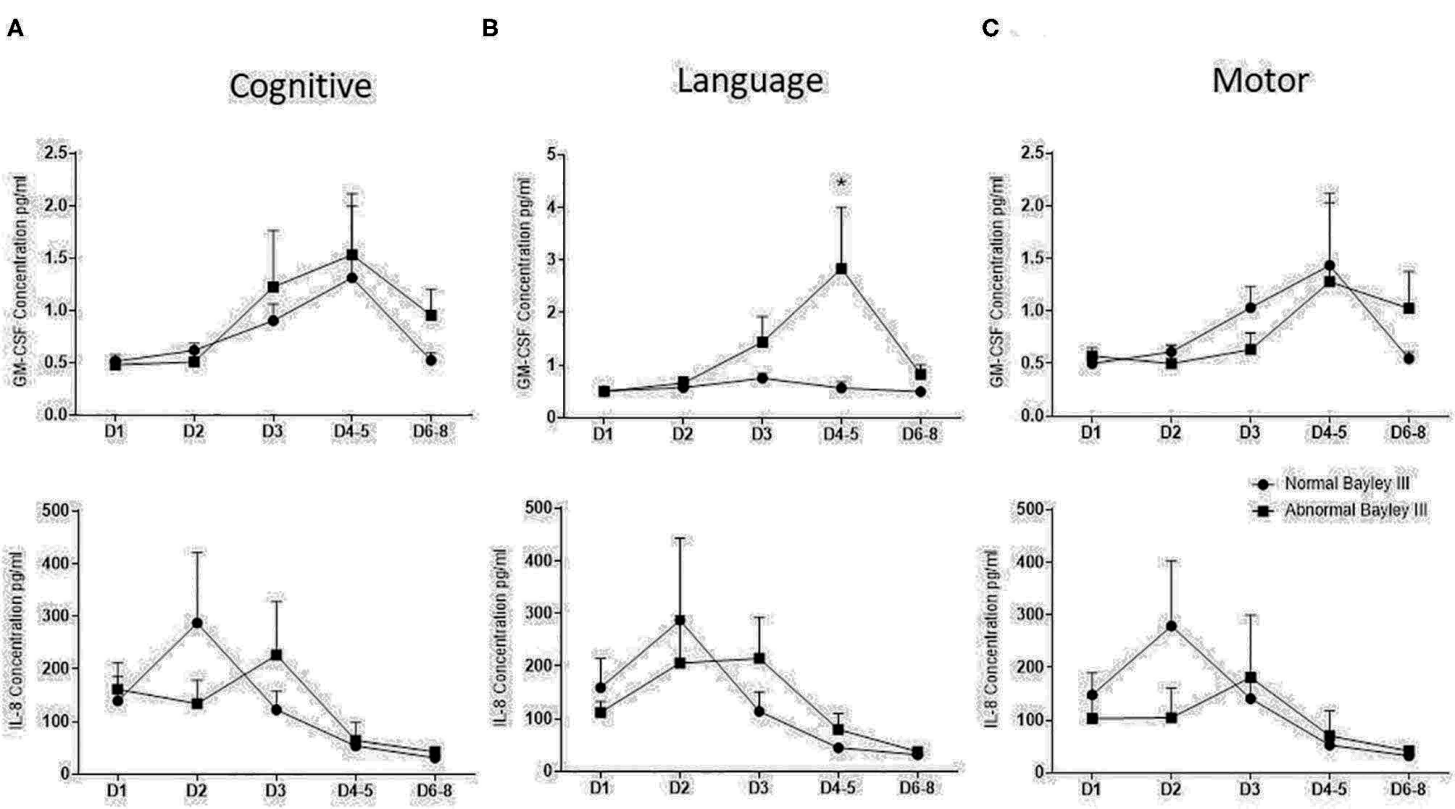
Correlation between GM-CSF and IL8 and neurodevelopmental outcome. Bayley developmental score was performed on NE infants at 2 years of age and correlated with GM-CSF and IL-8 concentrations obtained by duplex cytokine analysis. Graphs showing mean (± SEM) GM-CSF, at the top, and IL-8, at the bottom, concentration values between normal (circles) and abnormal (squares) Bayley score from NE neonates divided by cognitive **(A)**, language **(B)**, and motor **(C)** (*p < 0.05 using the Mann-Whitney test).

GM-CSF and IL-8 concentration did not correlate with cognitive and motor Bayley III scores. Elevated GM-CSF, but not IL8 concentration on days 4–5 of life correlated negatively with composite language (*p* = 0.0242) Bayley III scores at 2 years (Figure 1).

### Cerebrospinal Fluid Biomarker Data

Thirty-four infants had CSF samples collected with the following grades of encephalopathy: NE 0/I (*n* = 5); NE II/III (*n* = 29). CSF samples were taken on median (IQR) day 4 (1.8–5.0). Seventeen infants in this group required TH, 22 infants developed clinical seizures and 1 infant died. The median (IQR) for GM-CSF was 0.096 (0.06–0.28) pg/mL and for IL-8 was 236.02 (58.94–545.56) pg/mL. There were no significant associations found between either of the CSF biomarkers and TH, seizure occurence, NE grade, MRI result, Bayley-III score or mortality. There was no significant association between CSF biomarkers and the presence of severe brain injury on MRI imaging (grade 3 or 4 BG/W Barkovich Score).

## Discussion

In this study we found a significant association between decreased levels of GM-CSF on days 2 and 3 and abnormal MRI brain imaging. GM-CSF regulates cell growth and promotes proliferation of granulocytes and monocytes/macrophages (21). It has been previously reported that preterm infants who later develop CP have significantly lower levels of GM-CSF compared to controls (22) while term infants who are later diagnosed with CP have significantly higher levels of GM-CSF in their newborn heelprick samples compared to matched controls (23).

Marlow et al. (24) found that there was no detrimental effect of GM-CSF treatment on 2-year neurodevelopmental outcomes in a randomized control trial of preterm infants in which prophylactic GM-CSF was used to prevent sepsis. Similar to our cohort, Okazaki et al. (25) found no difference between levels of serum GM-CSF in severe NE infants who had TH (*n* = 5) compared to mild NE infants, who did not have TH (*n* = 5) and to controls (*n* = 4). In a previous cohort of infants with NE from our group we found increased GM-CSF on day 1 was associated with adverse outcomes (5). Despite the small numbers of infants who died in our current cohort, we found a significant association between elevated GM-CSF and IL-8 on day 1 of life with mortality. Spath et al. (14) have recently demonstrated that excess production of GM-CSF induced spontaneous brain inflammation and neurological dysfunction in an animal model by the production of ROS. We have previously found that neonatal neutrophils derived from umbilical cord blood had increased ROS and activation following GM-CSF treatment *in vitro* compared with either granulocyte colony-stimulating factor (G-CSF) or lipolysaccharide (8).

We found significant association between elevated day 2 IL-8 levels and grade II/III NE. This is in keeping with previous research showing elevation of IL-8 levels in asphyxiated term infants compared to healthy control infants on day 1 with levels equilibrating by day 4 of life (10, 11). In the first days of life in healthy newborns cytokines are stable and not significantly altered (26). Opposite to Bartha et al. (27) findings where IL-8 production in the brain was associated with abnormal neurodevelopmental outcomes in NE, we found that levels of IL-8 are not associated with abnormal neurodevelopmental outcomes, this discrepancy can be explained because in the Bartha study neurodevelopmental outcome was considered abnormal if the infant died, whereas in the present study these patients were excluded. High concentration of IL-8 in the CSF of term infants have been correlated with severe encephalopathy (27, 28). Term infants with abnormal outcomes in the short (11) and long-term using MRI-defined neuroabnormalities and adverse neurological outcome (27) have been reported to have significantly higher postnatal serum and newborn heelprick IL-8 levels compared to infants with normal outcomes. Nelson et al. (23) found significantly higher levels of IL-8 in the newborn heelprick samples of 31 infants who subsequently developed spastic CP compared to 65 term controls. IL-8 also appeared to correlate significantly with NE-induced seizures and may serve as a biomarker for earlier detection of brain damage in neonatal seizures (29). However, these studies were all done in the pre-TH era.

In this study, infants who underwent TH had significantly elevated serum and IL-8 levels on day 3 compared to those who did not receive TH. Our findings are supported by previous work by Jenkins et al. who found significantly higher levels of IL-8 in TH compared to normothermic infants with NE at most time points over the first 80 h of life. Infants following TH with better outcomes at 12 months showed uniform down regulation of IL-8 from their peak levels observed at 24 h to their nadir at 36 h (30).

GM-CSF and IL-8 are markers of brain injury in situations like stroke and neurodegenerative disorder. In this study we showed the association of serum GM-CSF and IL-8 levels with survival and brain damage, and the importance of the follow up of these cytokines in the first days of life in neonatal encephalopathy.

## Data Availability Statement

The raw data supporting the conclusions of this article will be made available by the authors, without undue reservation.

## Ethics Statement

The studies involving human participants were reviewed and approved by The Research Ethics Committee (REC) of The National Maternity Hospital. Written informed consent to participate in this study was provided by the participants' legal guardian/next of kin.

## Author Contributions

DS, AM, LK, and TS conceived the study and prepared the manuscript. DS and TS performed the laboratory experiments and analyzed the data. CO, WW, JM, and AO'N reviewed the final data analyses and contributed to the writing of the manuscript. MS performed the Bayley's assessments. VD is the radiologist who reported all cranial ultrasound and MRIs. EM supervised the design and execution of the study, performed the final data analyses, and contributed to the writing of the manuscript.

## Conflict of Interest

The authors declare that the research was conducted in the absence of any commercial or financial relationships that could be construed as a potential conflict of interest.

## Abbreviations

NE: neonatal encephalopathy
CP: cerebral palsy
VEGF: vascular endothelial growth factor
Epo: erythropoietin
IL-8: interleukin-8
GM-CSF: granulocyte-macrophage colony-stimulating factor
CSF: cerebrospinal fluid
TH: therapeutic hypothermia
BG: basal ganglia
W: watershed
BG/W: combined basal ganglia/watershed.
